# Draft Genome Sequences of Bacterial Strains Capable of Degrading Ulvan from the Green Alga Ulva ohnoi

**DOI:** 10.1128/MRA.01483-19

**Published:** 2020-01-16

**Authors:** Ratna Mondal, Miho Satoh, Kouhei Ohnishi

**Affiliations:** a The United Graduate School of Agricultural Sciences, Ehime University, Matsuyama, Ehime, Japan; b Institute of Molecular Genetics, Kochi University, Nankoku, Kochi, Japan; Georgia Institute of Technology

## Abstract

We present here the draft genome sequences of four marine bacterial strains which can use ulvan as their sole carbon source. We used ulvan extracted from the green alga Ulva ohnoi. Each bacterium contains a polysaccharide-utilizing locus, which is necessary for the complete degradation of ulvan.

## ANNOUNCEMENT

*Ulva* spp. (green algae) are distributed worldwide. Green algal blooms are responsible for coastal green tides (1). We may be able to use these macroalgae as a renewable resource (2). Ulvan is a complex water-soluble sulfated polysaccharide in the cell wall of *Ulva* spp. It is composed of α-linked l-rhamnose-3-sulfate, β-linked glucuronic acid, α-linked iduronic acid, and β-linked d-xylose. Ulvan polysaccharide and depolymerized oligoulvan can be used in various food, feed, biomedical, paint, and textile industries (3). Ulvan-utilizing bacteria may contain novel enzymes for the depolymerization and metabolization of alga-specific carbohydrates (4). The respective ulvan-degrading genes are usually located in gene clusters on the genome, which are called polysaccharide utilization loci (PULs) (5). The involved carbohydrate-active enzymes (CAZymes) are listed and classified in the CAZy database (http://www.cazy.org/) (6).

We collected natural Ulva ohnoi specimens, together with small conch snails and small shrimp, from Uranouchi Bay, Kochi, Japan, and cultivated them in artificial seawater under light (16 h/8 h day/night) at 25°C. The feces of the small animals digesting the green algae were collected and put into artificial seawater with ulvan extracted from *U. ohnoi* as the sole carbon source. Ulvan-utilizing bacteria were enriched at 25°C for 3 days and isolated on agar medium. We biochemically analyzed the activity of the ulvan lyase, which is the first enzyme needed for ulvan degradation, from ulvan-utilizing strain KUL17 (7). In order to identify PULs and elucidate the evolution of ulvan degradation enzymes, we determined the draft genome sequences of four bacterial strains, KUL10, KUL17, KUL42, and KUL49. Based on the partial nucleotide sequences of 16S rRNA genes, KUL10 was assigned as a Glaciecola sp., and three other strains were classified as Alteromonas spp. Chromosomal DNA was extracted from cultured cells of each strain in half-strength marine broth (BD Difco) using a Wizard genomic DNA purification kit (Promega) and fragmented with nebulization. After emulsion PCR, genome sequencing was performed using 454 GS Junior pyrosequencing with GS RunProcessor v2.5.3. The total number of reads for KUL10, KUL17, KUL42, and KUL49 were 164,694, 146,173, 145,967, and 141,656, with average read lengths of 476 bp, 443 bp, 476 bp, and 412 bp, respectively. The reads were assembled using GS De Novo Assembler v3.0. Gene annotation was conducted using the DFAST prokaryotic genome annotation pipeline (8). Default parameters were used for all software unless otherwise noted. Characteristics of the sequenced genomes are listed in Table 1.

**TABLE 1:** Characterization of the genome assemblies of 4 ulvan-utilizing bacteria

Strain	WGS accession no.	DRA accession no.	Genome size (bp)	GC content (%)	Coverage (×)	No. of contigs (longer than 200 bp)	*N*_50_ (bp)	CDSs^*a*^ (with protein)
KUL10	BGNG00000000	DRA009359	4,221,649	41.2	18	33	364,277	3,556
KUL17	BJKJ00000000	DRA009360	4,831,342	44.3	13	60	188,920	3,901
KUL42	BJKK00000000	DRA009360	5,000,727	42.8	13	48	233,207	4,102
KUL49	BJKL00000000	DRA009360	4,615,000	44.4	11	46	364,199	3,833

a CDSs, coding DNA sequences.

These draft genome sequences of ulvan-utilizing bacteria, along with the already published sequences of Nonlabens ulvanivorans (9), *Alteromonas* spp. (10), and a *Pseudoalteromonas* sp. (11), will open up possibilities for the usefulness of polysaccharide ulvan from *Ulva* spp.

### Data availability.

This whole-genome shotgun (WGS) project has been deposited at DDBJ/EMBL/GenBank. The versions described in this paper are the first versions. The read archives have been deposited in the DDBJ Sequence Read Archive (DRA). The WGS and DRA accession numbers are listed in Table 1.
